# Not too long, not too short: Goldilocks principle of eye size

**DOI:** 10.1371/journal.pgen.1008914

**Published:** 2020-07-15

**Authors:** Rachel W. Kuchtey

**Affiliations:** 1 Department of Ophthalmology and Visual Sciences, Vanderbilt University Medical Center, Nashville, Tennessee, United States of America; 2 Department of Molecular Physiology and Biophysics, Vanderbilt University, Nashville, Tennessee, United States of America; HudsonAlpha Institute for Biotechnology, UNITED STATES

For most of the world, normal vision and visual acuity are central to our lives and livelihoods. Abnormal vision is often equated with conditions such as cataracts or retinal degeneration, but increasingly recognized contributors to abnormal vision are serious disturbances in visual acuity caused by uncorrected refractive error. In 2017, the Vision Loss Expert Group projected that uncorrected refractive error will become a major cause of global vision impairment and blindness by 2020 [1]. Although refractive error such as nearsightedness (myopia) and farsightedness (hyperopia) is usually correctable with eyeglasses, high myopia and high hyperopia can result in significant vision loss, and even irreversible blindness, due to complications such as macular degeneration and glaucoma. Myopic macular degeneration gives rise to similar outcomes as does age-related macular degeneration, with a notable difference—the former affects patients of much younger age. At the other end of the refractive spectrum, high hyperopia commonly leads to angle closure glaucoma. Furthermore, extremely high hyperopia associated with nanophthalmos—a small eye caused by developmental abnormalities—almost always leads to angle closure glaucoma, for which surgical correction is challenging and often associated with severe complications [2]. Understanding the pathogenesis of refractive error is greatly needed to develop effective treatments.

High myopia or hyperopia is commonly caused by lengthening or shortening of axial length (AL) of the eye, respectively. If AL is too long, the sclera (the white outer layer of the eye) becomes stretched and thinned, predisposing to retinal detachment and degeneration of the retinal pigment epithelium. Conversely, if AL is too short, internal eye structures are crowded, and the sclera becomes thickened, predisposing to angle closure glaucoma and choroidal effusion.

Recent studies in *PLOS Genetics* provide new insight into the genes and gene interactions, that contribute to abnormalities in axial length and refraction, and, furthermore, represent an intersection between common and rare genetic disease. On the common side of the spectrum, myopia and hyperopia have been the subject of several genome wide association studies (GWAS). *TMEM98*, which encodes a widely expressed single transmembrane protein of unknown function [3,4], is one of several loci associated with high myopia in GWAS, but a definitive and mechanistic role for *TMEM98* in axial length has not been apparent from GWAS. On the rare side of the spectrum, however, deleterious coding alterations in *TMEM98* co-segregate with autosomal dominant nanophthalmos in large multigenerational families [5,6]. Intriguingly, nanophthalmos is the phenotypic opposite of high myopia, a paradox that is underscored by a report of 3 variants in *TMEM98* associated with high myopia in Chinese patients [7].

Nanophthalmos can be caused by genes other than *TMEM98*. In fact, the first nanophthalmos locus (*NNO1*) was mapped in 1998 [8], and last year, using the same family, Garnai and colleagues reported in *PLOS Genetics* that the underlying cause was a mutation in *MYRF* that leads to a C-terminal truncation [9]. *MYRF* encodes a transcription factor that regulates the production of myelin, especially for the optic nerve. But *MYRF* is also a gene in which different alleles lead to different phenotypes, including a syndromic condition with diaphragmatic hernia, cardiac defects, and urogenital abnormalities [10]. To better understand the role of *MYRF* and *MYRF* mutations in ocular development and disease, Garnai and colleagues added to their *PLOS Genetics* manuscript the investigation of a tissue-specific loss-of-function *MYRF* allele in mice, targeted to the developing retina and retinal pigment epithelium (RPE) [9]. Interestingly, these animals exhibited degeneration of the retina and RPE, but did not exhibit an alteration in axial length [9]. Now, mouse eyes are very different from human eyes, so absence of a shortened axial length in *Myrf* mutant mice is perhaps not so surprising [11]. On the contrary, *Myrf* mutant mice faithfully recapitulate the retinal abnormalities, which is perhaps not surprising since other genes identified in Mendelian nanophthalmos cases, such as *CRB1* and *BEST1*, have profound roles in the retina and RPE [12,13]. Although it has been postulated that the RPE, which is anatomically interposed between retina and sclera, plays a critical role in relaying signals from neural retina to sclera as an end target for regulation of axial length, the precise mechanism remains largely elusive and begs further investigation [14].

Cross and colleagues did just that in a study published in April 2020’s issue of *PLOS Genetics* [15]. The authors provide yet another surprising, but not unexpected finding, that mice lacking *Tmem98* have greatly enlarged eyes with long axial length. This study was built upon their previous investigation, which found that when two known missense mutations of *TMEM98* in human autosomal dominant nanophthalmos were engineered into mice, they did not have nanophthalmos, but instead developed retinal pathology [16]. They further confirmed the strong expression of *TMEM98* in RPE, consistent with previous reports [9]. To understand the mechanism of action, the investigators in the current study produced mice deficient for *Tmem98* in the RPE to avoid perinatal lethality caused by global knockout of the gene. Those mice developed retinal and RPE abnormalities as expected. More strikingly, they exhibited greatly enlarged eye size with compressed choroid and thin sclera, which could be detected as early as embryonic day 17.5 and persisted to adult life. The extremely thin sclera in the *Tmem98* mutant mice is likely the root cause of the extremely enlarged eye, yet electron microscopy failed to show obvious collagen fibril abnormalities, which is counterintuitive. It may even contradict the findings from surgical specimens of a nanophthalmos patient, as shown by Yamani *et al*. [17]. The full-thickness nature of the sclerectomy specimens of the human eye allowed detailed views of all three layers of sclera, in which the innermost layer- the lamina fusca, which is adjacent to the choroid and RPE- had the most pronounced collagen fibril abnormalities, highlighting a possible role of RPE in determining the thickened sclera phenotype in nanophthalmos. The lack of obvious abnormalities of collagen fibrils in the *Tmem98* conditional knockout mice could be due to the intrinsically thin nature of mouse sclera, perhaps highlighting the necessity of large animal models with similar eye size and anatomy to human eyes.

**Fig 1:**
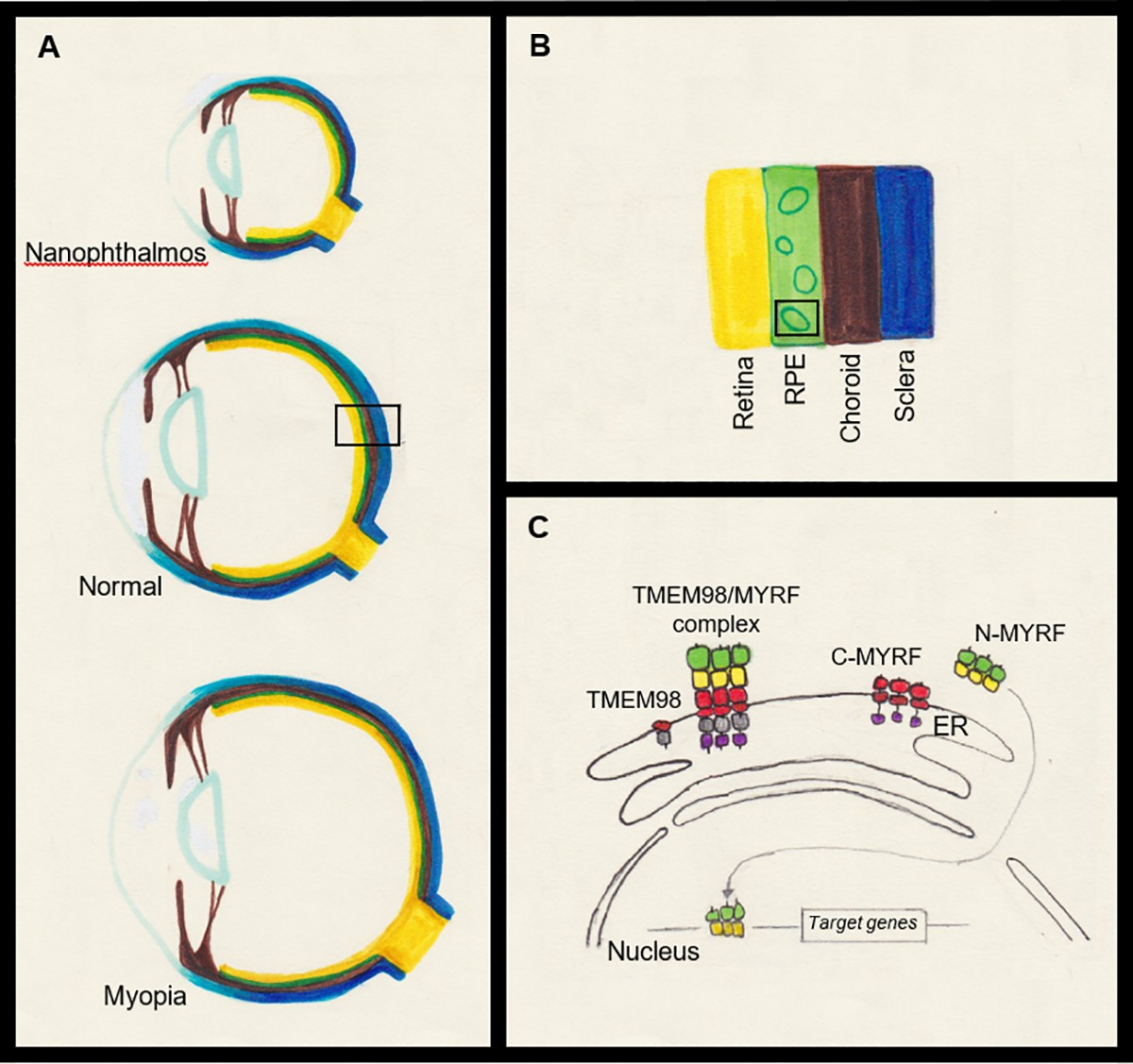
TMEM98 and MYRF in eye size regulation. **A.** Schematic drawing of the human eye. The eye size is an important factor in determining proper focus of images on the retina, and its regulation is complex. If the axial length is too short, hyperopic refractive error may occur. An extreme example is nanophthalmos (A, top). The opposite is myopic refractive error, which commonly results from long axial length (A, bottom). **B.** Close up view of posterior pole of normal size human eye (A, middle). Multiple layers of retina are condensed as a single layer and depicted in yellow. Retinal pigment epithelium (RPE) is in green, choroid in brown and sclera in blue. **C.** Close up view of molecular interaction between TMEM98 and MYRF in RPE cells. Mutations in *MYRF* cause autosomal dominant nanophthalmos in human patients, but conditional knock out of *Myrf* in mice does not show short axial length phenotype. Similar phenomena have been demonstrated in *TMEM98*. Furthermore, RPE specific conditional knock out of *Tmem98* in mice results in extremely enlarged eye size with compressed choroid and thin sclera. One of the interacting partners of TMEM98 is MYRF which is a membrane bound transcription factor. MYRF is inactive due to the failure of self-cleavage when bound by TMEM98. When TMEM98 is absent in the RPE, the N-terminal portion of MYRF (N-MYRF) is released without inhibition of TMEM98 and subsequently translocalized to the nucleus where it induces transcription of target genes.

To understand the molecular mechanism of action, the investigators performed an unbiased experiment and discovered MYRF as an interacting partner to TMEM98. This was an extremely important finding, but again came as no surprise for two main reasons: first, mutations of *MYRF* are known to cause autosomal dominant nanophthalmos in human patients [9]; second, Garnai and colleagues had previously discovered interaction of the same two proteins, prompting them to propose a new pathway for eye size regulation [9]. In addition, the interaction between those two proteins was already shown in oligodendrocytes in mice [18]. Because MYRF is a membrane-bound transcription factor activated by self-cleavage of the N terminus and nuclear translocalization of the C terminal portion, there likely exists a negative feedback loop between MYRF and TMEM98 (Fig 1). In fact, the study from Cross and colleagues bolsters that idea: TMEM98 inhibits the autoproteolytic cleavage of the N terminus of MYRF. When TMEM98 is lacking in the RPE, MYRF is ectopically activated and translocated to nuclei to activate transcription of downstream genes. It would be very interesting to know if *Myrf* mutant mice could rescue the phenotypes observed in *Tmem98* mutant mice. These experiments would shed more light on the regulation of eye size and could ultimately suggest therapeutic approaches to make the eye size just right–not too long, not too short.
